# Quantum Secure Direct Communication Technology-Enhanced Time-Sensitive Networks

**DOI:** 10.3390/e27030221

**Published:** 2025-02-21

**Authors:** Shiqi Zhang, Chao Zheng

**Affiliations:** 1 College of Science, North China University of Technology, Beijing 100144, China; 2 School of Energy Storage Science and Engineering, North China University of Technology, Beijing 100144, China; 3 Beijing Laboratory of New Energy Storage Technology, Beijing 100144, China

**Keywords:** quantum information, quantum technology, quantum-secure direct communication, time-sensitive network

## Abstract

Quantum information has emerged as a frontier in scientific research and is transitioning to real-world technologies and applications. In this work, we explore the integration of quantum secure direct communication (QSDC) with time-sensitive networking (TSN) for the first time, proposing a novel framework to address the security and latency challenges of Ethernet-based networks. Because our QSDC-TSN protocol inherits all the advantages from QSDC, it will enhance the security of the classical communications both in the traditional TSN- and QKD-based TSN by the quantum principle and reduce the communication latency by transmitting information directly via quantum channels without using keys. By analyzing the integration of QSDC and TSN in terms of time synchronization, flow control, security mechanisms, and network management, we show how QSDC enhances the real-time performance and security of TSN. These advantages enable our QSDC-TSN to keep the balance between and meet the requirements of both high security and real-time performance in industrial control, in a digital twin of green power and green hydrogen systems in distributed energy networks, etc., showing its potential applications in future quantum-classical-hybrid systems.

## 1. Introduction

Quantum secure direct communication (QSDC) is a complete new theory of communication, and it is firstly proposed in 2000 [1]. QSDC is able to achieve secure information transmission over quantum channels directly, of which this property makes it distinguishable and gives it advantages that other protocols do not have. Until now, it is developed very fast both in theory [1,2,3,4,5,6,7,8,9,10,11,12,13,14] and in experiment [14,15,16,17,18,19,20,21]. QSDC has a lot of remarkable characteristics. First, it has higher safety compared to quantum key distribution (QKD) as well as traditional TSN technology [14,22,23,24]. Based on the principles of quantum mechanics, QSDC uses resources such as quantum entangled states and single photons to transmit information. Based on the non-clonability of quantum states and quantum superposition, the leakage of information can be immediately detected even if an eavesdrover attempts to intercept the information [25]. During the communication process, if there is any eavesdropping, the communication parties can detect the eavesdropping in real time by measuring the quantum bit error rate (QBER) or using the Bell state measurement [17]. Second, there is no need to pre-assign keys. I.e., unlike the QKD, QSDC does not require a key to be established in advance, but instead transmits messages directly over a quantum channel, which reduces the security risk of potential intermediate links [2,26]. Third, it is suitable for high-capacity communication. QSDC can use multi-dimensional quantum states (such as multi-photon, entangled states) to transmit high-capacity information, such as each photon carrying multiple bits of information, which has a higher capacity than the traditional QKD [18,27,28].

At present, both theoretical works and experimental verifications of QSDC have been carried out extensively. Since it was first proposed in 2000, QSDC has undergone continuous progress from theoretical model to experimental implementation. In the theoretical aspect, the preliminary theoretical frameworks, such as the DL04 protocol and the two-step QSDC protocol, have been proposed and demonstrated for their effectiveness in quantum communication [1,2,3]. In order to improve the security of QSDC, some novel protocols have also been proposed, such as low-noise QSDC schemes based on quantum entanglement [4], and recently proposed measurement-device-independent QSDC and device-independent protocols [5,6]. These theoretical studies aim to eliminate the security risks caused by the detector or equipment vulnerabilities. In the experimental aspect, researchers improve the transmission distance and data transmission rate of QSDC. For example, the DL04 protocol in the experiment achieved a fiber-optic transmission of up to 18.5 kilometers and achieved a transmission rate of more than 100 kbps [18,19]. Experimental studies have shown that the QSDC protocol can achieve reliable and secure communication in the presence of noise and eavesdropping [20]. QSDC also can protect against potential security vulnerabilities during engineering [17]. In addition, a QSDC network prototype based on quantum entanglement has been experimentally validated [21].

Cao et al. proposed a simulation study of a two-step QSDC scheme based on EPR pairs, and verified the feasibility of quantum-secure direct communication through algorithms and visualization techniques [29]. In addition, quantum communication experiments based on free space also prove the feasibility of QSDC in open environments [30,31]. For applications, QSDC has been widely used in various fields. The primary application of QSDC is to enable high-security communications without key distribution, especially for institutes and industries that require the highest levels of secrecy during communications [1,25,32]. QSDC can be used to build multi-user quantum networks, supporting the development of large-scale quantum Internet through quantum entanglement and secure communication over very long distances [33,34,35]. In scenarios such as quantum cloud computing or data centers, QSDC can provide a physical level of security for data transmission and storage, preventing any eavesdropper from accessing or intercepting data in transit [36,37]. Protocols of QSDC have also been applied to the design of secure quantum auction systems to ensure the privacy of participants and the security of bidding information during the auction process [38,39]. Device-independent quantum-secure direct communication has also been proposed [40,41], and its channel capacity has increased dramatically [42,43], laying the foundation for the technology combination application of QSDC.

One of the traditional technologies based on classical physics and information science is time-sensitive networking (TSN). It can be seen as a set of Ethernet-based network technology standards designed to address the needs of highly reliable, low-latency, deterministic communication in industrial automation, vehicle networking, mobile forward, smart grid, and other application scenarios. The core idea of TSN is to provide end-to-end deterministic communication through accurate time-synchronization mechanisms, traffic scheduling, and bandwidth management, ensuring that data flows can be transmitted on time and achieve the required quality of service (QoS) [44,45]. TSN has clear characteristics and advantages. TSN ensures that the clocks of all devices in the network are highly synchronized by using an accurate clock-synchronization protocol, such as IEEE 802.1AS, enabling time-triggered and time-based scheduling communication [46,47]. In addition, the precise time protocol (PTP) is often used to improve synchronization accuracy [48]. TSN provides a set of traffic-scheduling mechanisms that can ensure the deterministic transmission of critical data flows, avoid network congestion and delay fluctuations, such as time-triggered scheduling (TAS) [49], priority flow control [50] and periodic queue scheduling [51], etc., to ensure the low-latency transmission of critical data flows. TSN achieves high reliability by using frame replication and elimination for reliability (FRER) technology to ensure that data streams are not interrupted during transmission due to packet loss or network failures [52]. TSN introduces a centralized network configuration (CNC) and a distributed network control mechanism that dynamically adjusts network resource allocation according to actual needs to achieve optimized network performance [53,54].

With its deterministic communications capabilities, TSN has become a key technology for industries as diverse as finance, automotive, manufacturing, healthcare, and avionics [55,56]. TSN plays an important role in industrial automation systems by providing low-latency, high-reliability communication guarantees for real-time control and equipment coordination [44,57]. TSN is used in vehicle-to-everything (V2X) to ensure communication synchronization and data flow management within the vehicle and on the shop floor, enabling autonomous driving and intelligent transportation systems [58,59,60]. TSN is used in smart grids to realize the real-time monitoring and data transmission of power systems, ensuring efficient and reliable communication between power equipment and management systems [61,62]. TSN is being combined with fifth-generation (5G) wireless communication technologies to create TSN-5G networks, which are seen as an excellent solution to industrial networking challenges [63]. TSN’s time synchronization has been improved to overcome competition, retransmission, and mobility issues in integrated 5G networks, achieving a breakthrough accuracy of one microsecond in industrial environments [64].

Although TSN can guarantee the low latency and high reliability of data, its security in network transmission still faces challenges. Traditional encryption technologies are struggling to cope with the growing number of cyber attacks, especially in critical industrial and control systems [65,66]. One attempt to improve the safety TSN, using quantum technology, is based on QKD protocols [65]. It can ensure absolute security in the communication process through the key distribution mechanism based on quantum mechanics, avoiding the risks that traditional encryption technologies such as key disclosure may face. Especially in the face of potential threats from quantum computing, it provides a reliable defense scheme [67]. It enhances the security of time synchronization. It provides a more secure encryption mechanism for time synchronization through quantum key distribution [68]. It supports real-time encryption for TSN. The combination of QKD technology and TSN provides the capability of real-time encryption, especially for data transmission of real-time-sensitive traffic. It can transfer encryption keys between TSN switches, and can dynamically adjust the key transfer rate to ensure that the data flow can be encrypted and decrypted in a timely manner, even in a high-load network environment [69]. This enhances the safety of TSN in complex industrial control environments. It also improves the stability and security of the entire network by providing secure encryption for control information, making communication between control nodes safe from attack [70]. Miao et al. proposed a traditional encryption scheme based on QKD technology for TSN time-sensitive service, synchronization information, and control information [71].

However, the fusion of QKD and TSN has shortcomings or defects. For example, it relies on classical communication channels to pass the key during key distribution, which means that this part of the communication can still be threatened by eavesdropping or man-in-the-middle attacks, as pointed in the previous work [67]. It has a limited key-generation rate and may not meet the needs of real-time encryption in large-scale and high-data scenarios, especially under high load conditions [70]. It has its limitations in highly dynamic environments. Channel attenuation and ambient noise in wireless and mobile networks have great influence on QKD. On one hand, QKD requires a complex key management system, especially in a multi-node network, whereby the difficulty of key management will increase significantly [67]. It has requirements for device compatibility. It requires specialized quantum key distribution equipment, while existing traditional TSN equipment may need to be upgraded or modified, resulting in higher costs [66]. On the other hand, the transmissions of keys in QKD protocols rely on classical communication channels that can be attacked.

QSDC overcomes the disadvantage in principle, because it transmits information directly through quantum channels only, and there is no need for classical channels to transmit keys. This property fundamentally eliminates the possibility of eavesdropper obtaining keys through classical channels, so it has advantages in improving communication security [72,73]. In this paper, we proposes the first QSDC-based TSN (QSDC-TSN protocol). Our proposal combines QSDC and TSN, and provides optimization and enhancement from multiple levels, including time synchronization, traffic scheduling, security mechanism, network management, a physical layer, and a link layer. Our QSDC-TSN takes advantages than the classical TSN and QKD-based TSN.

## 2. Results

### 2.1. Significance and Advantages of TSN and QSDC Fusion

QSDC allows information to be encrypted and decrypted at the same time, eliminating the bottleneck of separating key distribution and encryption process in traditional QKD. QKD still needs to encrypt and decrypt data using traditional encryption algorithm after key distribution, which may affect the transmission efficiency of TSN network. QSDC avoids this extra step, and the data are transmitted and encrypted at the same time, greatly improving the overall efficiency and reducing latency. The time-synchronization requirements of TSN are extremely high, especially in industrial control systems, where any delay may bring irreversible consequences, so as to better meet the real-time requirements of TSN.

The key-generation rate of QKD is often limited by physical devices and quantum-state generation and detection, which makes QKD may not meet the demand of real-time high-frequency key updating in large-scale, high-throughput TSN applications. For example, in high-speed data transmission scenarios such as industrial control and connected vehicles, data traffic, and encryption needs can change rapidly, and QKD may not be able to generate enough keys quickly enough to meet these needs. QSDC can transmit messages directly through quantum states, bypassing the limitation of key generation, and is suitable for high-rate and low-delay application scenarios.

QKD technology needs to establish a secure channel through key distribution, and then use the key for encrypted communication, which puts forward higher requirements for key management. For example, in large-scale industrial control systems, deploying and maintaining a large number of key management nodes can increase the complexity and maintenance cost of the system. QSDC can transmit information directly through quantum channel and ensure its security, which can reduce complex key management and distribution steps in TSN network, simplify network architecture, and be more suitable for large-scale distributed networks.

QSDC is more resistant to aggression. QSDC is resistant to quantum attacks and traditional man-in-the-middle attacks. In QKD, an attacker can influence the key distribution process by eavesdropping on some key communication nodes even if they cannot obtain the key. In QSDC, because the information is transmitted directly in the quantum layer, the attacker cannot obtain the information without being detected. Therefore, given that high security is required in time-sensitive networking, QSDC can provide a more robust defense mechanism to ensure the security of real-time sensitive data transmission.

QSDC demonstrates its unique advantages in the combination of TSN technology, especially in real-time, as well as security, a simplified architecture, an improved efficiency, and other aspects beyond QKD. Through the direct quantum communication mode of QSDC, the complexity of key management can be avoided, the network structure can be simplified, and the anti-attack methods can be enhanced, which makes it have great application potential in the real-time sensitive networks with high security requirements, such as in the industrial Internet and intelligent manufacturing domains.

### 2.2. Method of QSDC Combined with TSN

#### 2.2.1. Overall Implementation Method

The architecture of the combination of TSN and QSDC technology can be implemented through the following main modules:

1. Centralized network controller: Responsible for controlling and scheduling the data flow in the TSN, and communicating securely with the TSN switch through the QSDC controller.
2. QSDC controller: Responsible for generating and managing quantum channels, and securely transmitting communication information to TSN switch through quantum states.
3. PTP switching node: The PTP switching node is responsible for the secure transmission of time synchronization information and ensures that time-synchronization data is not tampered with through QSDC.
4. TSN switch: Accept CNC control signaling and data flow, and carry out safe time synchronization and data transmission through QSDC.

**Figure 1:**
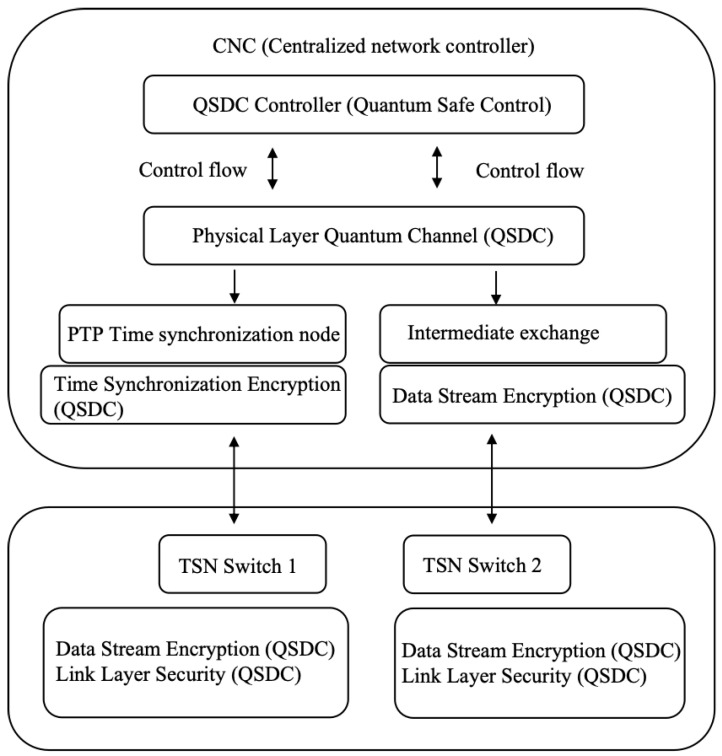
TSN architecture and QSDC technology combination diagram.

The TSN architecture and QSDC combination diagram is shown in Figure 1. The functions of each structure are as follows:

1. Centralized network controller: This node stands for centralized network controller, which communicates with TSN switch via QSDC controller.
2. QSDC controller: As an intermediate layer, it is responsible for quantum encryption transmission of communication between CNC, PTP switching nodes and TSN switches.
3. PTP switching node: Encrypts and transmits time synchronization information to ensure the security of PTP synchronization data.
4. TSN switches 1 and 2: Represent the switching devices in the network, which communicate with the CNC via QSDC and receive data streams.

**Figure 2:**
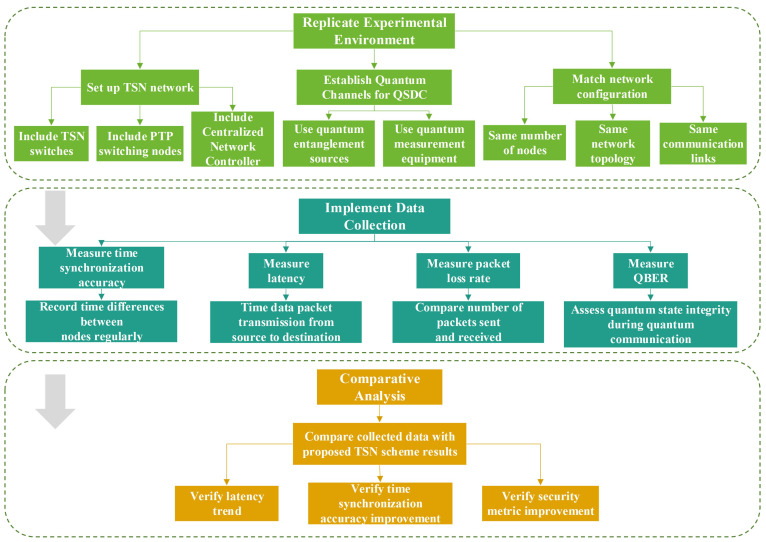
Flowchart of comparative experiments for TSN scheme based on QSDC.

To replicate or verify the comparisons made in the proposed TSN scheme, the recommended steps are as follows:

The flowchart of comparative experiments for TSN scheme based on QSDC is shown in Figure 2, in order to make the process more understandable. The first thing is that replicating the experimental environment. This includes setting up a TSN network with the necessary components such as TSN switches, PTP switching nodes, and a centralized network controller. For the QSDC part, establish quantum channels using appropriate quantum devices like quantum entanglement sources and quantum measurement equipment. Ensure that the network configuration, including the number of nodes, network topology, and communication links, is identical to that in the proposed scheme.

Data-collection mechanisms are implemented at various points in the network. Measure parameters such as time-synchronization accuracy, latency, packet loss rate, and security-related metrics like the QBER. For time synchronization, record the time differences between different nodes at regular intervals. To measure latency, time the transmission of data packets from the source to the destination. Packet loss rate can be calculated by comparing the number of packets sent and received. QBER can be measured during quantum communication to assess the integrity of quantum states.

The collected data are compared with the results presented in the proposed TSN scheme. They are checked to see if the measured latency in the replicated setup follows the same trend as the results in the conclusion. Similarly, the improvements are verified in terms of time-synchronization accuracy and security metrics.

By following these steps, the comparisons can be replicated and verified made in the proposed TSN scheme, validating the claimed advantages of integrating QSDC with TSN in terms of time synchronization, latency, security, and overall network performance.

We will start with the convergence of key components of the TSN architecture, especially in the areas of time synchronization, flow control, security mechanisms, and data transfer.

#### 2.2.2. The Combination of Time Synchronization Mechanism

**Figure 3:**
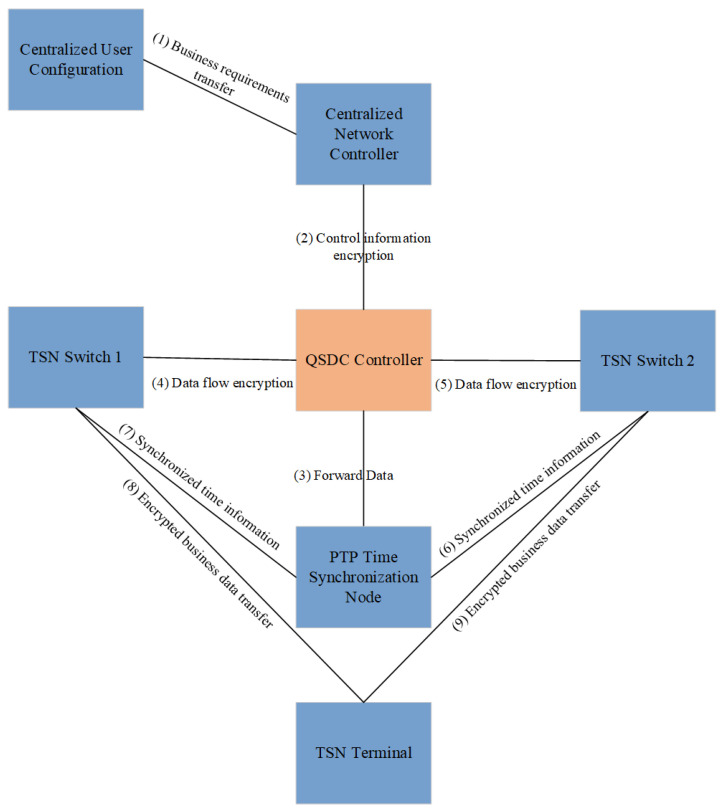
Time synchronization with QSDC diagram.

One of the key technologies of TSN is precise time synchronization, ensuring coordination between different devices and reducing latency. QSDC can be combined with the time-synchronization mechanism of TSN, especially in the process of transmitting synchronous information directly using quantum channel to encrypt and decrypt synchronous information. The time synchronization with QSDC diagram is shown in Figure 3.

The time synchronization of TSN depends on the PTP protocol, while QSDC can encrypt the timestamp information in PTP through the quantum state to ensure the data security during the time-synchronization process. The master clock nodes in CNC and TSN switches can encrypt their transmitted time-synchronization signals through QSDC, preventing eavesdropping from tampering with time information, and ensuring that each node in the network can obtain high-precision synchronization time. PTP time synchronization is encrypted and time-synchronization information (such as time stamps) in each TSN switch is encrypted via QSDC.

To improve TSN timeliness by QSDC, the time-synchronization information *I* needs to be encrypted and transmitted through a quantum channel. The QSDC encryption process for this data can be expressed as:

(1) $$C_{I}=E_{\mathrm{QSDC}}\left(I\right),$$

where $E_{\mathrm{QSDC}}$ represents the encryption operation, *I* is the original synchronization information, and $C_{I}$ is the encrypted synchronization data. By using QSDC, any attempt to intercept or alter the synchronization information during transmission is prevented, ensuring accurate synchronization across the network.

The total delay in a TSN network consists of several components, such as transmission delay ($T_{\mathrm{trans}}$), queuing delay ($T_{\mathrm{queue}}$), propagation delay ($T_{\mathrm{prop}}$), and encryption delay ($T_{\mathrm{enc}}$). The total delay $T_{\mathrm{total}}$ can be expressed as:

(2) $$T_{\mathrm{total}}=T_{\mathrm{trans}}+T_{\mathrm{queue}}+T_{\mathrm{prop}}+T_{\mathrm{enc}}.$$

In QSDC, the encryption delay $T_{\mathrm{enc}}$ is typically lower than traditional encryption methods, reducing the overall delay in the network. The introduction of QSDC thus helps maintain low latency and high synchronization accuracy, which is vital for TSN applications requiring real-time performance.

#### 2.2.3. The Combination of Flow Control and Scheduling Mechanism

**Figure 4:**
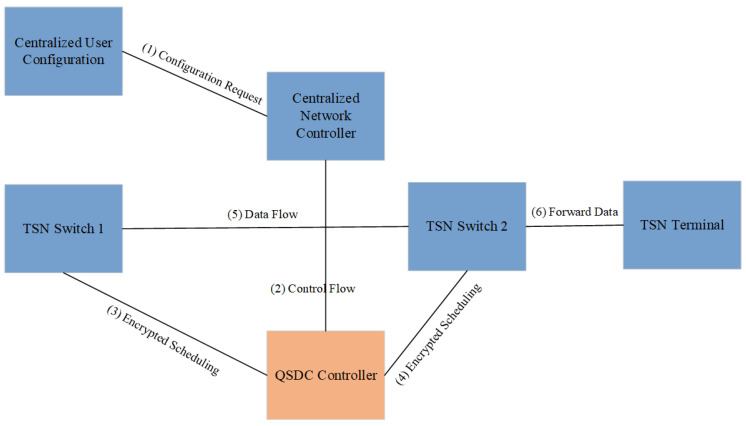
An illustration of the flow control and scheduling mechanism combined with QSDC.

TSN networks need to manage traffic with different priorities through accurate traffic scheduling to ensure that mission-critical data can be transmitted in a timely manner. An illustration of the flow control and scheduling mechanism combined with QSDC is shown in Figure 4.

In this process, QSDC can optimize and enhance the flow control and scheduling mechanism of TSN in the following aspects.

The encrypted traffic label transport in time-sensitive networking will be discussed. Each traffic packet has its own unique stream ID and priority information. These stream IDs and priority tags can be encrypted for transmission via QSDC, preventing attackers from analyzing traffic patterns to guess mission-critical flows in the network.

The scheduling policy in TSN is dynamically adjusted based on the real-time requirements of the business flow. In combination with QSDC, real-time encryption can be provided for dynamically scheduled packets, ensuring that data security can be maintained even if the data transmission path changes during scheduling. Specifically, QSDC can encrypt the path information and scheduling priority of each traffic packet, and only authorized nodes can decrypt and determine the processing priority of the packet.

#### 2.2.4. The Combination of Security Mechanisms

QSDC enhances security in multi-hop networks. In time-sensitive networking (especially in the cases of industrial control and large-scale networks), data need to be transferred through multiple switches. QSDC can ensure that at every jump point, data are encrypted through the quantum channel transmission; even if a certain jump point is controlled by an attacker, there is no way to decrypt and tamper with the data. This makes the multi-hop transport mechanism of TSN more secure and reduces the risk of data breaches due to a breach of the jump device.

In a multi-hop TSN network, assuming that the data pass through *N* switches, and QSDC encrypts the data at each hop, the encryption at each hop can be expressed as:

(3) $$C_{i}=E_{\mathrm{QSDC}}\left(D_{i}\right),\;\mathrm{for}\;i=1,2,\ldots ,N,$$

where $D_{i}$ is the original data at the *i*-th hop, and $C_{i}$ is the encrypted data at the *i*-th hop. By encrypting data at each hop, QSDC ensures that even if a switch is compromised, the data remain protected and cannot be altered or decrypted. This provides strong security for multi-hop transmission in TSN.

#### 2.2.5. The Combination of Network Management and Configuration

In the existing network architecture of TSN, CNC is the core of centralized management of the entire network, responsible for managing traffic scheduling and controlling the behavior of individual switches. We can strengthen the security of the entire network by introducing a QSDC Controller between the CNC and TSN switches.

There is a quantum-secure encryption of a centralized controller. The CNC needs to broadcast network configuration information to every node in the TSN, which often contains key network configuration and control instructions. QSDC can ensure the security and integrity of communication directly through the quantum channel without the need for traditional centralized key management. The configuration information sent by the CNC can be quantum-encrypted at the same time as the transmission. A QSDC controller needs to be deployed in the CNC, and the QSDC controller is responsible for providing the CNC with a quantum channel and transmitting the control signal through the quantum state. All the scheduling information of the CNC is encrypted by the QSDC and sent to the TSN switch.

Network configurations are encrypted using QSDC as follows:

(4) $$C_{\mathrm{conf}}=E_{\mathrm{QSDC}}\left(\mathrm{conf}\right),$$

where $C_{\mathrm{conf}}$ is the encrypted configuration data, conf is the original configuration information, $E_{\mathrm{QSDC}}$ is the encryption operation. By eliminating the need for traditional key management systems, QSDC reduces the bandwidth and overhead required for secure configuration updates, simplifying the network management process.

The configuration transmission delay is part of the total network management delay $T_{\mathrm{conf}-\mathrm{total}}$ is the encrypted synchronization data, which can be expressed as:

(5) $$T_{\mathrm{conf}-\mathrm{total}}=T_{\mathrm{trans}}+T_{\mathrm{enc}}+T_{\mathrm{queue}},$$

where the lower encryption delay of QSDC $T_{\mathrm{enc}}$ leads to faster configuration updates, improving the responsiveness and efficiency of network management.

#### 2.2.6. Combination of the Physical Layer and Link Layer

The introduction of QSDC can provide higher security at both the physical layer and the link layer. In a TSN network, the communication between the physical layer and the link layer is vulnerable to man-in-the-middle attacks or other security threats at the network level.

The physical layer is the base layer of network communication and is responsible for transmitting the raw bit stream over a physical medium, such as fiber optics or radio waves. After the introduction of QSDC technology, the security of physical layer is realized through the quantum channel, and QSDC technology can provide the quantum security of physical layer. This means that even if an attacker attempts to eavesdrop or interfere at the physical layer, the eavesdropping behavior will be detected immediately due to the non-clonability of quantum communication and the nature of the quantum superposition state, thus preventing any kind of physical layer attack.

The link layer is responsible for data transmission between two directly connected nodes and is the first line of data security in the network. QSDC can replace the traditional link layer encryption mechanism to realize point-to-point quantum-secure communication. With QSDC technology, the link layer encrypts all data flows between the switch and the PTP time-synchronization node.

## 3. Discussion

Classic TSN technology ensures real-time data transmission with low latency and high bandwidth through strict traffic management and a time-synchronization mechanism. In classic TSN, traditional encryption methods such as AES and RSA are used to ensure the security of communication data. Time synchronization in the network is carried out through the IEEE 802.1AS protocol, ensuring that the clocks of all devices are consistent, thus enabling real-time application support. In order to meet the demand of real-time data transmission, TSN also uses traffic queuing, bandwidth reservation, and other technologies for traffic scheduling, and ensures the timely transmission of important data streams through the priority mechanism. However, the encryption technology of classical TSN relies on traditional methods and has potential security risks.

The combination of QSDC and TSN provides a new solution for real-time and high-security applications. The traditional TSN relies on classical encryption methods and key management systems to secure data. But compared with QSDC, these methods are more vulnerable to classical channel attacks such as eavesdropping and man-in-the-middle attacks. QSDC transmits encrypted data directly through quantum channels, eliminating the dependence on classical channels and fundamentally improving network security. Unlike QKD, QSDC is able to encrypt and decrypt data at the same time as they are transmitted, significantly reducing latency, which is critical for TSN applications where real-time requirements are extremely high. QSDC simplifies the network architecture, avoids the complexity of traditional key distribution and management, and greatly reduces the deployment and maintenance costs of TSN networks, especially in large-scale application scenarios such as intelligent manufacturing and industrial Internet of Things. In addition, QSDC provides stronger security in multi-hop transmissions, ensuring that even in complex network environments, data remain encrypted and protected from tampering or theft. At the same time, QSDC also optimizes time synchronization in TSN, ensuring that the clocks of all devices in the network remain precisely synchronized, further enhancing the stability and reliability of the system. We sorted out the key differences between QSDC and QKD in TSN applications in Table 1.

In general, the combination of QSDC and TSN not only solves the problem of traditional network security and a real-time scenario, but also improves the scalability and flexibility of the network, which is especially suitable for real-time applications with high throughput, low latency, and high security, such as industrial automation, intelligent manufacturing, and autonomous driving systems. We sorted out the detailed advantages of QSDC enhanced time-sensitive networking in Table 2.

## 4. Conclusions

The integration of QSDC with TSN significantly enhances network security, efficiency, and real-time performance, making it particularly suitable for applications with high security and real-time requirements, such as industrial control. Unlike QKD, QSDC transmits information directly through quantum channels, eliminating the risk of eavesdropping over classical channels. It also enables simultaneous encryption and decryption during transmission, reducing latency and complexity. Additionally, QSDC bypasses the limitations of QKD in key generation speed, making it suitable for high-rate, low-latency applications. It simplifies network architecture by reducing the need for complex key management and provides stronger resistance to attacks. When combined with TSN, QSDC enhances security and optimizes efficiency across multiple areas, including time synchronization, flow control, multi-hop transmission, and network management. This integration of QSDC-TSN offers significant potential for improving performance in networks such as industrial Internet and smart manufacturing. Future applications of our QSDC-TSN can be expected in distributed energy networks, digital twins of green power and green hydrogen systems, etc.

## Figures and Tables

**Table 1:** Key differences between QSDC and QKD in TSN applications.

Feature	QSDC	QKD	TSN (Classic Technology)
Encryption Process	Simultaneous encryption and data transfer	Separate key distribution and encryption	Uses classical encryption methods (e.g., AES, RSA)
Key Management	No need for key management infrastructure	Requires key distribution system	Requires relatively complex key management infrastructure
Security	Resilient to both quantum and classical attacks	More vulnerable to attacks	Resilient to classical attacks but vulnerable to quantum attacks in future contexts
Latency	Low latency due to simultaneous encryption	Higher latency due to key distribution	Higher latency, and can be affected by encryption and routing overhead
Scalability	Highly scalable, no key update required	Limited scalability, requires frequent key updates	Scalable but requires strong network infrastructure and can face scalability issues with high traffic

**Table 2:** Detailed advantages of QSDC enhanced time-sensitive networking.

Aspect	Impact of QSDC on TSN
Security	Enhanced by quantum encryption at each hop
Latency	Reduced through simultaneous encryption and transmission
Scalability	Can handle high-throughput applications without key distribution bottleneck
Time Synchronization	Secures PTP synchronization with encrypted timestamps
Network Complexity	Simplified by eliminating traditional key management systems

## Data Availability

Data are contained within the article.

## Abbreviations

The following abbreviations are used in this manuscript:

QSDC	Quantum-secure direct communication
TSN	Time-sensitive network
QBER	Quantum bit-error rate
QKD	Quantum key distribution
QoS	Quality of service
PTP	Precise time protocol
TAS	Time-triggered scheduling
FRER	Frame replication and elimination for reliability
CNC	Centralized network controller
V2X	Vehicle-to-everything
5G	Fifth-generation
